# Insolubilization of Chestnut Shell Pigment for Cu(II) Adsorption from Water

**DOI:** 10.3390/molecules21040405

**Published:** 2016-03-28

**Authors:** Zeng-Yu Yao, Jian-Hua Qi, Yong Hu, Ying Wang

**Affiliations:** 1 Key Laboratory for Forest Resources Conservation and Use in the Southwest Mountains of China, Ministry of Education, Southwest Forestry University, Kunming 650224, China; jhqi1977@163.com (J.-H.Q.); huyong760925@163.com (Y.H.); 2 Faculty of Science, Southwest Forestry University, Kunming 650224, China; yingwang_2009@163.com

**Keywords:** *Castanea*, heating, heavy metal, melanin, removal

## Abstract

Chestnut shell pigment (CSP) is melanin from an agricultural waste. It has potential as an adsorbent for wastewater treatment but cannot be used in its original state because of its solubility in water. We developed a new method to convert CSP to insolubilized chestnut shell pigment (ICSP) by heating, and the Cu(II) adsorption performance of ICSP was evaluated. The conversion was characterized, and the thermal treatment caused dehydration and loss of carboxyl groups and aliphatic structures in CSP. The kinetic adsorption behavior obeyed the pseudo-second-order rate law, and the equilibrium adsorption data were well described with both the Langmuir and the Freundlich isotherms. ICSP can be used as a renewable, readily-available, easily-producible, environmentally-friendly, inexpensive and effective adsorbent to remove heavy-metal from aquatic environments.

## 1. Introduction

Industrialization, which can be advantageous for human beings, can alter the natural flow of materials and introducing novel chemicals into air, soil and water. Heavy metals, a kind of essential chemical to modern living, are produced in many industries and universally used. Effluents from their producing industries and wastes from their use lead to heavy-metal contamination of water bodies. These heavy metals can be detrimental to a variety of living species: copper is essential to organisms at trace concentrations but toxic, even lethal, at high concentrations [1].

Many biopolymers are known to trap heavy metals strongly, and using such biopolymers as adsorbents to remove toxic metal contaminants has been a hot research topic due to their renewable nature. Melanin is a kind of natural pigment widely presented in all large taxa from both prokaryota and eukaryota. It is formed by condensation of phenolic or indolic substances, and many other metabolites of the organisms, such as proteins and carbohydrates, can become incorporated into melanin. Therefore, most of the melanin biopolymers are deemed heterogeneous and irregular in composition and structure [2]. Melanin has high complexing abilities towards various heavy metal ions and plays a significant role in detoxifying such ions for the organisms. It has hydroxyl, carboxyl, and amine groups, which are potential sites for metal-ion binding [3,4]. Thus far, studies on the interaction between metal ions and melanin have mainly been for medicinal purposes. Although developing melanin as adsorbents for heavy metal removal is infrequently reported, the existing information demonstrates its great potential in the wastewater treatment. Bridelli and Crippa [5] pointed out that the affinity of melanin towards metal ions is very strong, comparable to the most efficient materials employed in decontamination and recovery techniques. In a recent work by Chen *et al.* [6], the adsorption yields of heavy metals on squid melanin were high and stable, with the maximum capacities of 0.93 and 0.65 mmol·g^−1^ for Cd(II) and Pb(II), respectively. The melanin biopolymers obtained from *Aureobasidium pullulans* and *Cladosporium resinae* were efficient for copper biosorption. The copper biosorption on *A. pullulans* obeyed the Freundlich and the Langmuir isotherms whereas the biosorption on *C. resinae* obeyed the BET isotherm [7].

Chestnut is one of the most popular nuts in the world. According to the report of the Food and Agriculture Organization (FAO) [8], the world’s total production of chestnut was 2.01 million tons in 2013. Chestnut shell, including pericarp (fruit coat) and testa (seed coat), is a byproduct from the chestnut processing industry. Considering the shell is about 10% of the weight of the unshelled nut, a huge amount of chestnut shell is produced by the industry. This plentiful byproduct has not yet been efficiently utilized and it remains a waste material. The shell residue contains around 15% brown pigment that has been identified as the melanin [9]. Some researchers have endeavored to develop such melanin as a colorant or antioxidant for food use [9,10,11]. However, its toxicology and safety evaluation have not been fulfilled, and as yet it has not been practically applied. Our research group makes efforts to look for new applications of the pigment which have never been involved in food chains. Being melanin, it has the potential for adsorptive removal of heavy metals from wastewater. However, we found that the pigment was partly dissolved in neutral and acidic and fully dissolved in basic aqueous media. Hence, it is hardly used as an adsorbent in its original state, and modification is needed to insolubilize or fixate it. We have developed two techniques to achieve this goal. One is crosslinking the pigment with formaldehyde to form water-insoluble resins [12], and the other is immobilizing it onto silica gel to get hybrid adsorbents [13]. In the present study, we developed another technique—insolubilizing the pigment by heating. To elucidate the conversion of the chestnut-shell pigment (CSP) to the insolubilized chestnut-shell pigment (ICSP), the samples were characterized by thermal gravimetric analysis (TGA), differential scanning calorimetry (DSC), Fourier transform infrared spectroscopy (FT-IR), elemental analysis, and solid-state ^13^C nuclear magnetic resonance (^13^C-NMR). A batch method was used to evaluate the Cu(II) adsorption performance of ICSP.

## 2. Results and Discussion

### 2.1. TDA and DSC of CSP

**Figure 1:**
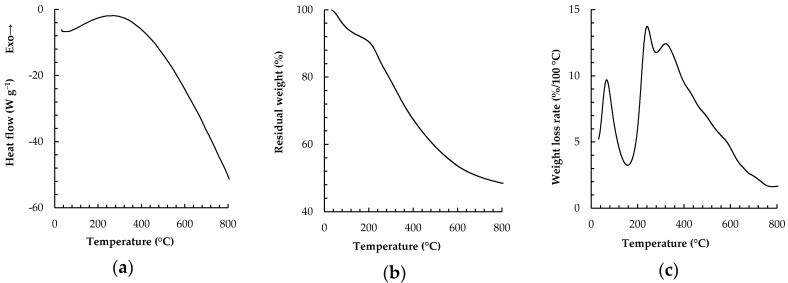
(**a**) Differential scanning calorimeter; (**b**) thermal gravimetry; and (**c**) differential thermal gravimetry of chestnut shell pigment (CSP).

The conversion of CSP to ICSP by heating was characterized by DSC and TGA. The typical DSC, TG, and DTG curves are exemplified in Figure 1. The negative heat flow that appears in the whole DSC curve (Figure 1a) indicates that the thermal processing was an endotherm at all the scanned temperatures (32–800 °C). The absolute value of the heat flow decreased with the temperature until 294 °C, and then it increased progressively with further heating up to 800 °C.

As shown in Figure 1c, the mass was lost in three stages, peaking around 68, 251 and 322 °C. The weight loss was about 7.8% at the first stage (32–157 °C), 12.7% at the second (157–294 °C), and 31.6% at the last (294–800 °C) (Figure 1b). At the first stage, the mass loss and the endotherm were mainly associated with evaporation of free and chemically bound water. At the second stage, they were mainly attributed to the condensation of carboxyl and hydroxyl groups. According to the result from a TGA/FT-IR coupling measurement done by He *et al.* [14], the gas product from CSP was mainly water moisture, without CO_2_, below 220 °C. So we selected this temperature to insolubilize CSP. At the last stage, they might be ascribed to the pyrolysis degradation of the CSP skeleton. The decrease in heat flow from the beginning to 294 °C may be due to gradual exhausts of the water and the functional groups for the condensation, and the subsequent upsurge of heat flow probably results from an increased energy demand for the further skeleton degradation.

### 2.2. Solubility of ICSP in the Aqueous Media

**Figure 2:**
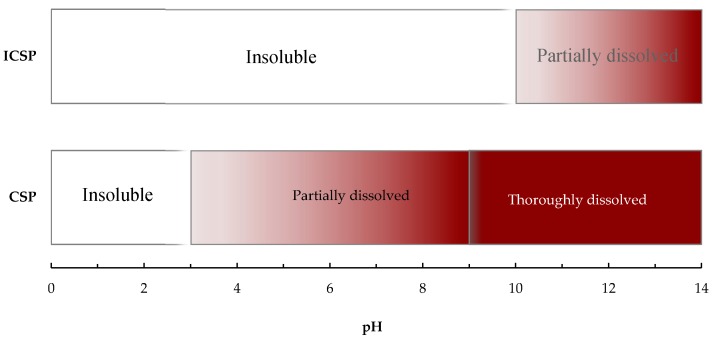
Solubility tendency of CSP and insolubilized chestnut shell pigment (ICSP) in aqueous media at various pH.

The solubility tendency of CSP and ICSP in aqueous media is shown in Figure 2. An increase in the pH values was favorable to dissolving CSP and ICSP. The thermal treatment improved the dissolving resistance of the biopolymer against water. As shown in Figure 2, CSP started to dissolve at about pH 3, whereas ICSP was insoluble until pH 10. Therefore, ICSP can be used as a solid phase in aqueous media at pH below 10.

### 2.3. ^13^C-NMR of CSP and ICSP

**Figure 3:**
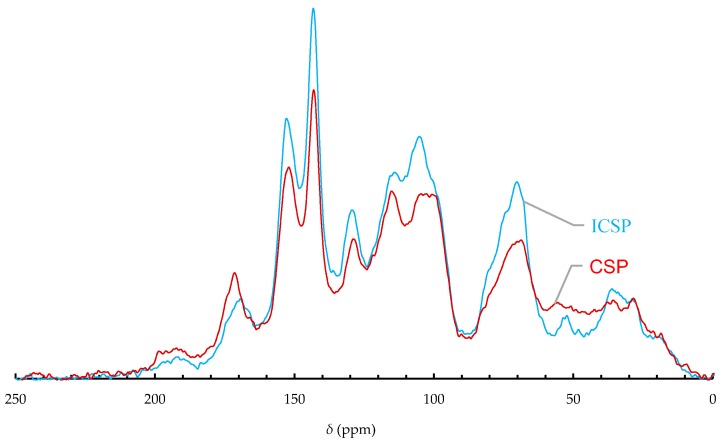
^13^C-NMR spectra of CSP and ICSP.

Application of solid-state ^13^C-NMR technique allows quick qualitative investigation of structural change in CSP after the thermal insolubilization. The CP/MAS ^13^C-NMR spectra of CSP and ICSP are shown in Figure 3. The spectra show broad resonances, which resulted from many overlapped signals due to the heterogeneous and paramagnetic nature of the melanin [9]. Various molecular fragments in CSP and ICSP can be identified from the spectra, and their relative abundance was estimated from the spectra by integrating the area under the corresponding peaks in the total spectral area. The chemical shift regions, the molecular fragments, and the relative abundance are listed in Table 1. The insolubilization appeared to reduce the intensities of alkyl, amino, methoxy, carboxyl, and carbonyl groups. Melanin biopolymers are capable of binding a variety of chemicals, including proteins and carbohydrates [2]. Some probabilities could attribute to these losses: (1) Degradation of aliphatic moieties of CSP; (2) Volatilization or decomposition of aliphatic compound bound on CSP; (3) Maillard reactions between the bound carbohydrates and proteins and caramelization of the carbohydrates; and (4) the loss of the carboxyl groups, probably due to degradation removal or reaction with other groups such as hydroxyls to form ethers. There were intensity increases of phenolic hydroxyls, of aromatic rings, and of C–O groups in carbohydrates, alcohols, and ethers. These increases probably resulted from the reduction of the functional groups mentioned above. Polar functional groups in melanin, which form hydrogen bonds with water, play important roles in its dissolution in water. Amongst them, acidic groups such as carboxyls and phenolic hydroxyls are essential for dissolving melanin in an alkaline aqueous phase. The insolubilization of CSP may mainly be due to the loss of carboxyl groups, which are one kind of adsorption site in melanin for heavy-metal ions. As seen from the ^13^C-NMR spectrum of ICSP (Figure 3) and Table 1, not all of these sites were lost after the insolubilizing treatment.

### 2.4. Elemental Composition of CSP and ICSP

To verify the conversion of CSP to ICSP, further elemental analysis was performed and the results are listed in Table 2. In contrast to the increase of the O content, the contents of the other elements went down after the heating treatment. The increases in the O content and its molar ratio to C might be attributed to the oxidation of the sample and the loss of the other elements. The decreases in the H content and its molar ratio to C probably, as mentioned in Section 2.3, resulted from the dehydration and the loss of carboxyl groups, of aliphatic moieties in the CSP skeleton, and of bound aliphatic compounds, which are abundant in hydrogen. The nitrogen and the sulfur in phyto-melanin are mainly from bound proteins, so the decreases in both contents of N and S and their molar ratios to C in the sample indicate the proteins were significantly changed during the thermal treatment.

### 2.5. FT-IR of CSP and ICSP

**Figure 4:**
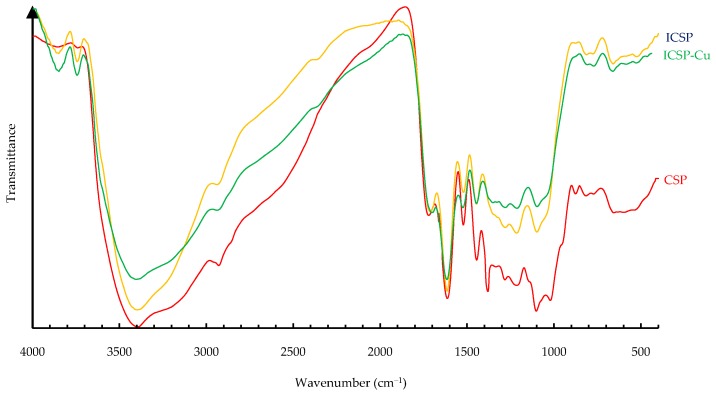
FT–IR spectra of CSP, ICSP and copper loaded ICSP.

To examine the structural changes from CSP to ICSP and further to copper-loaded ICSP, FT-IR spectra were recorded and are presented in Figure 4. The FT-IR spectra exhibit many absorption bands. The broad bands at 3380 cm^−1^ primarily are attributed to O–H stretching and secondarily to N–H stretching. The small cluster bands around 2930 and 2850 cm^−1^ correspond to stretching of aliphatic C–H groups. The peaks at 1710 cm^−1^ are due to C=O stretching of COOH. The bands centered at 1610 cm^−1^ are ascribed to aromatic C=C stretching and COO^−^ symmetric stretching. The absorbance at 1520 cm^−1^ assigns to N–H deformation, C–N stretching, and aromatic C=C stretching. The peaks at 1450 cm^−1^ represent aliphatic C–H deformation. The absorption at 1380 cm^−1^ is considered as OH deformation, C–O stretching of phenolic OH groups, and symmetrical stretching of COO^−^ groups. The bands in the range of 1240–1200 cm^−1^ can be attributed to C–O stretching and OH deformation of COOH, C–O stretching of phenols. The absorbance peaks in the range of 1110–1000 cm^−1^ may correspond to skeletal vibrations of aliphatic groups. By comparison of absorbance intensities for ICSP with those for CSP, we found some decrements in intensities of the bands representing, to some extent, different groups, which include the bands at 3380 and 1520 cm^−1^ with respect to hydroxyl and amino groups, at 1450 cm^−1^ and 1110–1000 cm^−1^ for aliphatic groups, and at 1380 cm^−1^ and 1240–1200 cm^−1^ for COOH. These pieces of evidence suggest that such functional groups were lost after suffering the heating, agreeing with the results derived from the ^13^C–NMR data and the element contents.

After the copper loading on ICSP, the peak intensities at 3380 cm^−1^ and 1240–1200 cm^−1^ were reduced, indicating that carboxyl, amino and hydroxyl groups are probable binding sites on ICSP for copper ions. Those groups are also considered as potential sites for metal-ion binding on other melanin [3,4].

### 2.6. Adsorption Kinetics

**Figure 5:**
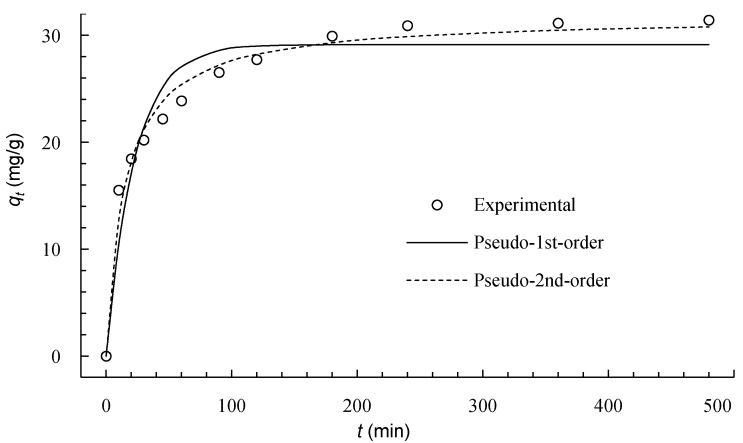
Adsorption kinetics of Cu(II) onto ICSP.

The dynamic adsorption process of Cu(II) onto ICSP was studied, and the result is illustrated in Figure 5. The amount of copper adsorbed on ICSP increased with the prolongation of the contact time and reached an equilibrium within 240 min. The adsorption process showed evidence for a multiphasic kinetics. At the initial rapid stage (0–20 min), the adsorption was fast and contributed to the major equilibrium uptake. This was probably mainly caused by the external adsorption of Cu(II) onto the ICSP surface. The second one was a slow adsorption stage (20–60 min), whose contribution to the copper adsorption was relatively smaller. At this stage, the copper was adsorbed gradually, and the process was controlled by a diffusion rate. Finally (60–240 min), the binding sites were being used up and the copper ions in the solution were extremely low when the adsorption system was reaching equilibrium. The adsorption achieved equilibrium within 8 h, as seen from Figure 5.

Some kinetic models are widely used to illustrate mechanisms of adsorption processes. In order to investigate the kinetics of the adsorption process of Cu(II) onto ICSP, two famous kinetic models were employed in this study. These were the pseudo-first- and the pseudo-second-order equations.

The pseudo-first-order model can be written as following equation [15]:

*q_t_ = q_e_* (1 – exp(–k_1_·*t*)) (1)

where *k*_1_ (min^−1^) is the adsorption rate constant, and *q_e_* (mg·g^−1^) and *q_t_* (mg·g^−1^) are the amounts of copper adsorbed at equilibrium and at time *t* (min), respectively.

The pseudo-second-order kinetic model is represented as [16]:

*q_t_* = *k*_2_·*q_e_*^2^·*t*/(1 + *k*_2_·*q_e_·t*) (2)

where *k*_2_ (g·mg^−1^·min^−1^) is the rate constant in the pseudo-second-order adsorption process, and *q_t_*, *q_e_* and *t* are the same as the definitions in Equation (1).

By non-linearly fitting the experimental data to Equations (1) and (2), the *k*_1_, *k*_2_, and *q_e_* in the models were determined and are reported in Table 3. The plot of *q_t_* as a function of *t* and its fitting curves are shown in Figure 5. The curve for the second-order kinetic model shows good agreement with the experimental values, which was confirmed by the quite high value of the determination coefficient, *R*^2^, listed in Table 3. The *q_e_* calculated from this model is closer to the experimental *q*_e_. So the pseudo-second-order model is more reasonable than the pseudo-first-order model to predict the dynamic adsorption of Cu(II) onto ICSP. The applicability of the pseudo-second-order model indicates that the adsorption was based on chemical reactions [16].

### 2.7. Adsorption Isotherms

Many adsorption isotherms, from fundamental or empirical viewpoints, have been used to study heavy metal removal from aqueous solutions. The Langmuir and the Freundlich isotherms have proven useful in engineering applications and been extensively used. Here, we restrict our interest to these two models to fit the equilibrium data of copper adsorption on ICSP.

The Langmuir adsorption model is valid for single-layer adsorption, assumes as a process occurring on homogeneous binding sites on adsorbents, and is generally expressed by the following non-linear equation [17]:

*q_e_ = q_m_·K_L_·C_e_*/(1 + *K_L_·C_e_*) (3)

where *q_m_* (mg·g^−1^) and *q_e_* are the monolayer adsorption capacity of the adsorbent and the amounts of copper adsorbed at equilibrium, respectively; *K_L_* (L·mg^−1^) is the Langmuir constant providing information on free energy of the adsorption process; and *C_e_* (mg·L^−1^) is the copper concentration in the solution at equilibrium.

The Freundlich isotherm is an empirical equation on the basis of assuming that the adsorption process occurs on the adsorbent surface with heterogeneous binding sites. The Freundlich model can be expressed by the following equation [18]:

*q_e_ = K_F_·C_e_*^1/*n*^ (4)

where *q_e_* and *C_e_* are the same as the definitions in Equation (3), and *K_F_* (mg^1−1/*n*^·g^−1^ L^1/*n*^) and 1/*n* (−) are empirical parameters correlated to the adsorption capacity and to the adsorption intensity, respectively.

**Figure 6:**
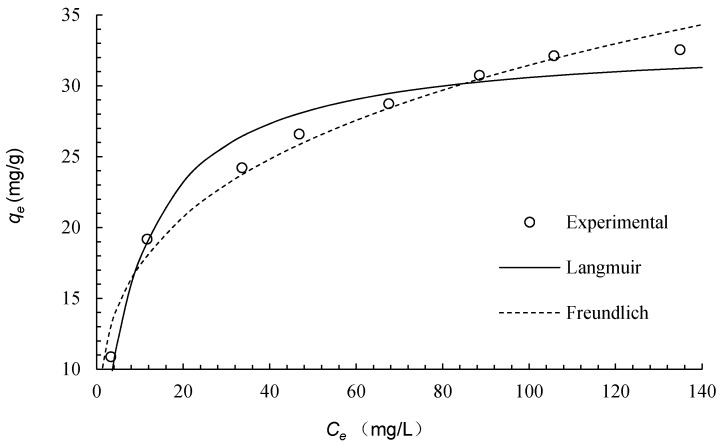
Adsorption isotherms of Cu(II) onto ICSP.

The Langmuir and the Freundlich isotherm plots for the adsorption of Cu(II) onto ICSP are reported in Figure 6. Parameter values for these models were calculated by a nonlinear regression method and are given in Table 4. The high *R*^2^ values for both isotherm models suggest that the experimental equilibrium data can both be described with the Langmuir and the Freundlich equations. Based on the plots of Figure 6 and the *R*^2^ values in Table 4, the Freundlich seems to fit the equilibrium data slightly better than the Langmuir. The 1/*n* value of the Freundlich model can be used to indicate favorability of adsorption systems. The 1/*n* value below 0.1 represents irreversible, in the range 0.1–0.5 easy, 0.5–1 moderately difficult, and above 1 poor adsorption characteristics [19,20,21]. As seen from Table 4, the 1/*n* is 0.259, which indicates that the copper ions are favorably adsorbed by ICSP.

Very few studies have been carried out on heavy metal adsorption by melanin or its derivatives [4,5,6,7], and no studies have reported on insolubilization of melanin biopolymers. Zhou *et al.* [12] used the resin prepared from CSP cross-linked with formaldehyde to adsorb copper and got an adsorption capacity of 29.8 mg·g^−1^. Su *et al.* [13] loaded CSP onto SiO_2_ to make the hybrid adsorbent, which gave copper adsorption capacity of 18.9 mg/g. ICSP gave a better copper adsorption capacity (33.2 mg·g^−1^), as shown in Table 4, than Zhou’s resin and Su’s hybrid adsorbent and even better than some commercial cation-exchange resins such as 1200H, Amberjet 1500H and Ambersep 252H [12,13,22,23]. Humic acid is a natural compound derived from biomass and has been commercially marketed as a sequestering agent. As a macromolecular phenolic substance, it is similar to melanin in structure physicochemical properties, and potential applications [9]. The insolubilization technique has also been employed for adsorbent preparation from humic acid. ICSP gives much better copper adsorption capacity than the insolubilized humic acid [24].

The insolubilization process does not involve difficult and time-consuming chemical treatments. Therefore, the adsorbents are also easier to prepare by this method than by Zhou’s and Su’s methods. In addition, CSP is obtained from agricultural waste biomass. Compared to some commercial resins for heavy-metal removal, ICSP gives some advantages, such as resource renewability and environment compatibility. As a result, ICSP presents a bright prospect for application in heavy-metal loaded wastewater treatment.

## 3. Materials and Methods

### 3.1. Materials

Chestnut fruits (*Castanea mollissima* Blume) were purchased from a local market in Kunming, China. All chemicals used were of analytical grade. Cu(II) solutions for adsorption experiments were prepared by diluting a stock solution (10,000 mg·L^−1^), which was prepared by dissolving a weighted quantity of Cu(NO_3_)_2_·3H_2_O in pure water.

### 3.2. Preparation and Insolubilization of CSP

In our previous study [12], we expressed a preparation procedure for CSP. The chestnut fruits were peeled manually. The obtained shells were crushed and sieved through 4 mm meshes. The scraps were soaked in 0.2 mol·L^−1^ NaOH aqueous solution at a liquid–solid ratio of 15 mL·g^−1^ at 50 °C for 12 h. The pigment extract was separated from the mixture through filter cloth, and the solid residue was re-extracted in the same manner. After vacuum filtration through filter paper, the extract was acidified to pH 2 with HCl, stood for 12 h at room temperature (~25 °C), and centrifuged at 3500 rpm for 10 min. The gel-like precipitate was collected and put in a refrigerator at −5 °C for 12 h. Then, the frozen precipitate was thawed at the room temperature and centrifuged to get CSP pellets. The product was washed twice with dilute HCl of pH 3. Finally, the pigment was dried at 50 °C. To insolubilize it, the dried CSP was heated in a temperature controlled oven at 220 °C for 3 h according to pre-experiments.

### 3.3. TGA and DSC

For TGA and DSC analyses, around 3 mg of dry CSP sample was scanned with a Mettler-Toledo TGA/DSC 1 instrument (Schwerzenbach, Switzerland) from 32 to 800 °C at a heating rate of 10 °C/min under nitrogen atmosphere.

### 3.4. Solubility Test

The solubility test was conducted to determine the pH range at which ICSP can be used as a solid phase in aqueous media without any hesitation of solubility. Five milligrams of CSP or ICSP were mixed with 5 mL water with a definite pH in a test tube. After 24 h agitation at the room temperature, the mixture was centrifuged at 5000 rpm for 10 min and absorbance of the supernatant was measured at 400 nm on a Shimadzu 1900 UV-Vis spectrophotometer (Kyoto, Japan).

### 3.5. ^13^C-NMR Analysis

The solid-state ^13^C-NMR spectra were acquired on a Bruker AVANCE III 400 MHz spectrometer (Rheinstetten, Germany) operating at a frequency of 100.369 MHz. About 0.3 g solid samples were confined in a zirconium oxide rotor with an external diameter of 7 mm and spun at 5 kHz in a magic-angle spinning (MAS) probe. Cross-polarization (CP) contact time and pulse delay were 2.0 ms and 1 s, respectively. Accumulation was repeated 10,000 times. The spectra were processed offline and integrated using the MestRe-C software (version 4.9.9.9, Mestrelab Research, Santiago de Compostela, Spain). For each sample, the integration was performed on specific spectral regions, as reported in Section 2.3. Each integrated area is the average value of three independent calculations, and the standard error is less than 5% of the mean.

### 3.6. Elemental Analysis

The C, H, N, S and O contents of the CSP and ICSP were determined using an Elementar Micro Vario elemental analyzer (Hanau, Germany).

### 3.7. FT-IR Spectroscopy Analysis

The FT-IR spectroscopy was carried out on a Bruker Tensor 27 FT-IR spectrometer (Karlsruhe, Germany). The spectra were obtained by grinding each sample with KBr at a mass ratio around 1:400 and pressing the mixture into a disc.

### 3.8. Adsorption Experiments

All adsorption experiments were conducted by a batch process on a temperature-controlled shaker at 120 rpm. In kinetic experiments, 0.05 g ICSP was added to a 25 mL copper solution of 200 mg·L^−1^ with initial pH 6 (according to pre-experiment results and adjusted with NaOH and HNO_3_) and shaken at 30 °C for different contact time. To determine equilibrium adsorption isotherms, the mixtures of 0.1 g ICSP and 50 mL Cu(II) solutions with different concentrations (25–200 mg·L^−1^) were shaken for 24 h at 30 °C. After the adsorption, the adsorbent was removed by a microporous membrane with a pore size about 1.2 μm on an injector, and the residual copper concentrations in the solutions were determined by a Perkin-Elmer AA-100 flame atomic absorption spectrometer (Norwalk, CT, USA). The adsorbed amount of copper, *q* (mg·g^−1^), was calculated according to Equation (5).

*q* = (*C*_0_ − *C_f_) V*/*m* (5)

where *C*_0_ (mg·L^−1^) and *C_f_* (mg·L^−1^) are the initial and the final Cu(II) concentrations in the aqueous phase, respectively, *V* (L) is the solution volume, and *m* (g) is the mass of sorbent used.

All the adsorption experiments were carried out in triplicate. Values used in the calculations were arithmetic averages of the experimental data. All the nonlinear model parameters were evaluated by nonlinear regression analysis using the SPSS 21 software.

## 4. Conclusions

CSP was converted to ICSP by a heating treatment, and ICSP is insoluble in aqueous media at the pH range of 1–10. The treatment lead to the dehydration, the loss of carboxylic groups, aliphatic moieties in CSP skeleton, and bound aliphatic compounds. ICSP was used to adsorb Cu(II) from water, and the carboxylic, amino and hydroxyl groups on ICSP are binding sites. Kinetic behavior of the adsorption obeys the pseudo-second-order rate law, and the equilibrium adsorption data can be well described with both the Langmuir and the Freundlich isotherms. ICSP has a good copper adsorption performance and can be used as a renewable, readily-available, easily-producible, environmentally-friendly, inexpensive and effective adsorbent to remove heavy metals such as copper from aquatic environments.

## Figures and Tables

**Table 1:** Relative abundances of different carbon in CSP and ICSP estimated from ^13^C-NMR data.

δ (ppm)	Molecular Fragments	Percentage (%)	
		CSP	ICSP
0–47	saturated alkyl carbons (CH, CH_2_, and CH_3_)	12.0	9.9
47–60	Carbons of amino groups and/or –O–CH_3_ moieties	4.8	2.9
60–111	C–O groups of carbohydrates, alcohols, and ethers	31.2	36.4
111–148	Carbons of aromatic rings	30.6	32.4
148–164	Car–O of phenols	11.2	11.7
164–188	Carboxyl carbon atoms	7.2	4.9
188–220	Carbonyl carbons (quinone, aldehyde and ketone)	3.0	1.8

**Table 2:** Elemental compositions and molar ratios of CSP and ICSP.

Samples	Elemental Composition (wt %)					Molar Ratio			
	N	C	H	S	O	N/C	H/C	S/C	O/C
CSP	1.39	52.0	5.54	0.53	40.6	0.023	1.28	0.004	0.586
ICSP	1.04	51.5	4.64	0.39	42.5	0.017	1.08	0.003	0.618

**Table 3:** Kinetic parameters for Cu(II) adsorption onto ICSP.

*q_e,exp_* (mg·g^−1^)	Pseudo-First-Order			Pseudo-Second-Order		
	*q_e,cal_* (mg·g^−1^)	*k*_1_ (10^−3^ min^−1^)	*R*^2^	*q_e,cal_* (mg·g^−1^)	*k*_2_ (10^−3^ g·mg^−1^·min^−1^)	*R*^2^
32.6	29.1	44.0	0.917	31.7	2.10	0.979

*q**_e,exp_*** is the experimental value of *q_e_*, *q**_e,cal_*** the corresponding theoretical value of *q_e_* derived from the kinetic models.

**Table 4:** Isotherm parameters for Cu(II) adsorption onto ICSP.

Langmuir			Pseudo-Second-Order		
*q_m_* (mg·g^−1^)	*K_L_* (L·mg^−1^)	*R*^2^	1/*n*	*K_F_* (L^1/n^·mg^1−1/n^·g^−1^)	*R*^2^
33.2	0.116	0.985	0.259	9.56	0.991

## References

[1] Stern B.R. (2010). Essentiality and toxicity in copper health risk assessment: Overview, update and regulatory considerations. J. Toxicol. Environ. Health. A.

[2] Plonka P.M., Grabacka M. (2006). Melanin synthesis in microorganisms—Biotechnological and medical aspects. Acta Biochim. Pol..

[3] Zdybel M., Chodurek E., Pilawa B. (2015). Free radicals in ultraviolet irradiated melanins and melanin complexes with Cd(II) and Cu(II)—EPR examination. J. Appl. Biomed..

[4] Hong L., Simon J.D. (2007). Current understanding of the binding sites, capacity, affinity, and biological significance of metals in melanin. J. Phys. Chem. B.

[5] Bridelli M.G., Crippa P.R. (2008). Theoretical analysis of the adsorption of metal ions to the surface of melanin particles. Adsorption.

[6] Chen S.G., Xue C.H., Wang J.F., Feng H., Wang Y.M., Ma Q., Wang D.F. (2009). Adsorption of Pb(II) and Cd(II) by squid *Ommastrephes bartrami* melanin. Bioinorg. Chem. Appl..

[7] Gadd G.M., Rome L. (1988). Biosorption of copper by fungal melanin. Appl. Microbiol. Biotechnol..

[8] Food and Agriculture Organization of the United Nations Faostat. http://faostat3.fao.org/browse/Q/QC/E.

[9] Yao Z., Qi J., Wang L. (2012). Isolation, fractionation and characterization of melanin-like pigments from chestnut (*Castanea mollissima*) shells. J. Food Sci..

[10] Qi J.-H., Yao Z.-Y., Wang L.-H. (2012). Comparison of antioxidant activities of crude pigments extracted from chestnut shell by ethanol and alkali. Sci. Technol. Food Ind..

[11] Yu X., Huang K., Zou J. (1997). Studies on characteristics and application of the pigment from chestnut shell. Chem. Ind. Forest Prod..

[12] Zhou M., Su P., Qi J.-H., Hu Y., Yao Z.-Y. (2014). Double-catalyzed base-acid synthesis of chestnut shell pigment resin cross-linked with formaldehyde. Appl. Mech. Mater..

[13] Su P., Zhou M., Qi J.-H., Kan H., Yao Z.-Y. (2014). Synthesis and copper sorption of chestnut-shell-pigment/SiO_2_ composite. Adv. Mater. Res..

[14] He L., Wang X., Xu R. (2007). Study on the thermal decomposition of the pigment extracted with water from chestnut shell. Food. Mach..

[15] Lagergren S. (1898). About the theory of so-called adsorption of soluble substances. K. Sven. Vetenskapsakad. Handl..

[16] Ho Y., McKay G. (1998). Kinetic models for the sorption of dye from aqueous solution by wood. Process Saf. Environ. Prot..

[17] Langmuir I. (1918). The adsorption of gases on plane surfaces of glass, mica and platinum. J. Am. Chem. Soc..

[18] Freundlich H. (1906). Concerning adsorption in solutions. J. Phys. Chem..

[19] Duong D.D. (1998). Adsorption Analysis: Equilibria and Kinetics.

[20] Ayranci E., Hoda N. (2005). Adsorption kinetics and isotherms of pesticides onto activated carbon-cloth. Chemosphere.

[21] Zhang B., Yang R., Zhao Y., Liu C.-Z. (2008). Separation of chlorogenic acid from honeysuckle crude extracts by macroporous resins. J. Chromatogr. B.

[22] Rengaraj S., Kim Y., Joo C., Choi K., Yi J. (2004). Batch adsorptive removal of copper ions in aqueous solutions by ion exchange resins: 1200H and IRN97H. Korean J. Chem. Eng..

[23] Rengaraj S., Yeon J.-W., Kim Y., Jung Y., Ha Y.-K., Kim W.-H. (2007). Adsorption characteristics of Cu(II) onto ion exchange resins 252H and 1500H: Kinetics, isotherms and error analysis. J. Hazard. Mater..

[24] Gezici O., Kara H., Yanık S., Ayyildiz H.F., Kucukkolbasi S. (2007). Investigating sorption characteristics of copper ions onto insolubilized humic acid by using a continuously monitored solid phase extraction technique. Colloids Surf. A.

